# The use of nonpharmacological interventions for chronic pain treatment in community-dwelling older adults with a certified need for care

**DOI:** 10.1186/s12877-024-05317-2

**Published:** 2024-09-04

**Authors:** Daniela Koios, Ronny Kuhnert, Dagmar Dräger, Arlett Wenzel, Reinhold Kreutz, Andrea Budnick

**Affiliations:** 1 Institute of Medical Sociology and Rehabilitation Science, Charité - Universitätsmedizin Berlin, Charitéplatz 1, 10117 Berlin, Germany; 2 Institute of Clinical Pharmacology and Toxicology, Charité - Universitätsmedizin Berlin, Charitéplatz 1, 10117 Berlin, Germany

## Abstract

**Background:**

Chronic pain is a major health issue and rapid population ageing exacerbates the burden to health systems in countries like Germany. Nonpharmacological interventions (NPIs) are essential in pain care and the prioritization of active NPIs is emphasized in guidelines. This paper examines the utilization of NPIs for chronic pain management in community-dwelling older adults with a certified need of care in Berlin, Germany.

**Methods:**

Cross-sectional data was collected through standardized face-to-face surveys with older adults (≥65 years), using validated instruments (e.g., Brief Pain Inventory), and structured lists for NPI utilization. Categorization into active and passive NPIs was performed through a literature-based, iterative process by an interdisciplinary team. For not normally distributed data, non-parametric tests were used as appropriate. Logistic regression was conducted for multivariate analysis.

**Results:**

In total, 250 participants were included in this analysis (aged 65-104, x̅ = 81.8, 68.8% female). Most (92%) use NPIs for chronic pain management: 85.6% use active NPIs, 50.4% active movement and only 5.6% use solely passive approaches. Most common NPIs are distraction, thermotherapy/compresses, and physiotherapy. The odds of utilizing physiotherapy are three times higher for those with high educational status when compared to those with low education while those with low educational status had higher odds of using thermotherapy/compresses.

**Conclusions:**

In our sample, most community-dwelling older adults with a certified need of care use active NPIs for chronic pain management with about half using active movement approaches. Considering the high vulnerability of this population, physiotherapy (in the form of therapeutic exercise) is a particularly appropriate intervention, and it was the third most frequent NPI in our sample. However, there is a social gradient in the utilization of physiotherapy for chronic pain management which might be rooted in issues around awareness, appeal, and access to such measures. It is important to take socioeconomic differences into account when planning the care for older chronic pain patients but also when designing research or user-friendly guidelines for this target group.

**Trial registration:**

Ethical approval from the Ethics Committee of Charité - Universitätsmedizin Berlin (EA1/368/14) and study registration with the Central Study Register (ZSR no. 20009093).

Chronic pain is a major cause of reduced quality of life and disability. Nonpharmacological interventions (NPIs) for chronic pain management are an important part of holistic treatment concepts since they can improve levels of pain intensity and related interference and may reduce or, in some cases, even eliminate the need for analgesic medication [1–5]. While surgical approaches can also be considered ‘nonpharmacological’ [6], this paper solely focusses on non-invasive NPIs. In a European cross-sectional study from 2006, two thirds of adults with chronic pain indicated the use of some kind of NPI [7] and a study from Australia found that more than one third of primary care patients with chronic pain use at least one form of NPI [8]. In a more recent European study, Morrissey, O'Neill [9] focus on older adults with chronic musculoskeletal pain and their utilization of complementary and alternative medicine (CAM) approaches. The authors [9] found that about one third (33.5%) of pain-affected participants utilized CAM (e.g., massage or osteopathy) and 28.3% used physiotherapy in the previous year. Overall, research focussing on NPI utilization for pain management specifically in older adults is scarce and trials and systematic reviews assessing the effectiveness of NPIs often lack statistically significant results [e.g., 5, 10–14] while chronic pain management guidelines are often “informed by expert opinion, not high-quality evidence” [3].

The American Chronic Pain Association and the Stanford University Division of Pain Medicine [15] define “active interventions” as requiring the person with pain to “use their mind and/or body as part of the treatment” whereas “passive interventions […] can be received without any active participation by the person with pain”. The authors [15] further state that “activity is always part of treating chronic pain”. Examples for active measures include exercise, therapeutic movement programs or distraction “with pleasurable activities” while massage or acupuncture, but also analgesic medications, are considered “passive” [15]. In the context of NPIs, recommendations and guidelines for primary care providers often emphasize that active measures should be prioritized over passive ones [6, 16, 17] and the latest German primary care guideline for chronic pain clearly states that active movement approaches should always form the basis of nonpharmacological chronic pain management [18]. In addition, some guidelines specifically recommend physical therapy/physiotherapy (from here on referred to as “physiotherapy”) as an intervention, for example for chronic low back pain [19] or for knee osteoarthritis [20], but also for chronic non-cancer pain in general [18].

The principle of prioritizing active approaches aligns with current evidence [e.g., 4, 21, 22]; however, it remains unclear how this is put into practice by older, community-dwelling persons who have a certified need of care. While all age groups can be affected by pain that persists for three months or longer, prevalence generally increases with age [23, 24]. Similarly, functional disabilities and neurological disorders become more common as we age, resulting in a higher burden of co-morbidities in older adults [25, 26]. Once such impairments lead to a particular need for support in daily activities (e.g., personal care or household management), the German social system offers an assessment to acknowledge a certified need of care [27]. The extent to which support is needed is structured by care grades, ranging from 1 (i.e., slight impairment of independence and ability) to 5 (i.e., most severe impairment with special care needs). When a level of impairment is certified by the responsible authority, the person is entitled to certain financial and/or care services. In 2022, more than five million people in Germany had such a certified need of care, 79% of whom are 65 years or older [28].

The majority (84%) of those with a certified need of care live at home (as opposed to in a nursing home or assisted living facilities) and almost one million German residents aged over 65 years receive home care through ambulatory nursing services [28]. Leiske et al. [29] report a chronic pain prevalence of 68.5% in adults with a certified need of care who receive ambulatory care services in Germany. Compared to the general population, this especially vulnerable group is affected by co-morbidities to a greater extent which may affect general mobility and other pre-requisites for active interventions such as exercise. Overall, adults aged 60 or over who suffer from chronic pain were found to be less physically active than those without chronic pain [30]. Whether less physical activity leads to chronic pain or vice versa is elusive, but older people’s beliefs and the fear avoidance model seem to play an important role in the context of activity levels and chronic pain [3, 31–33]. In the context of current guidelines and these specific characteristics of our target group, it appears especially relevant to distinguish not only between the passive or active nature of each NPI, but to further differentiate whether an intervention involves physical movement or not.

This article presents a descriptive analysis of NPI utilization for chronic pain management in community-dwelling older adults with a certified need of care in Berlin, Germany, and we analysed potential group differences based on sociodemographic markers. In addition, we seek to examine whether the utilization of certain NPIs (or groups of NPIs) is associated with differences in the perceived acceptability of people’s pain situation, their pain levels, and/or pain-related interferences. To our knowledge, there are no German studies focussing on the utilization of NPIs in this specific target group. In the light of Germany’s rapidly ageing population [34] information regarding chronic pain management for community-dwelling older adults becomes increasingly relevant. By describing the use of NPIs and potential differences in this specific group, the results may be useful for researchers, primary care providers and policy makers alike as they may inform future recommendations for research and practice.

## Methods

The analysis for this paper is based on a data set from the cross-sectional ACHE study (Development of a Model for P**A**in Management in Older Adults Re**C**eiving **H**ome Car**E**). From 2017-2018, older adults (≥65 years) with chronic pain (defined as pain persisting or recurring for ≥3 months) were interviewed using a standardized questionnaire in their homes in Berlin, Germany (*n *= 355). Ethical approval was obtained from the Ethics Committee of Charité - Universitätsmedizin Berlin (EA1/368/14) and all participants (or their legally authorized representatives) gave written informed consent. The study was registered with the Central Study Register (ZSR no. 20009093). Further details regarding the study design were described previously [35].

**Fig. 1:**
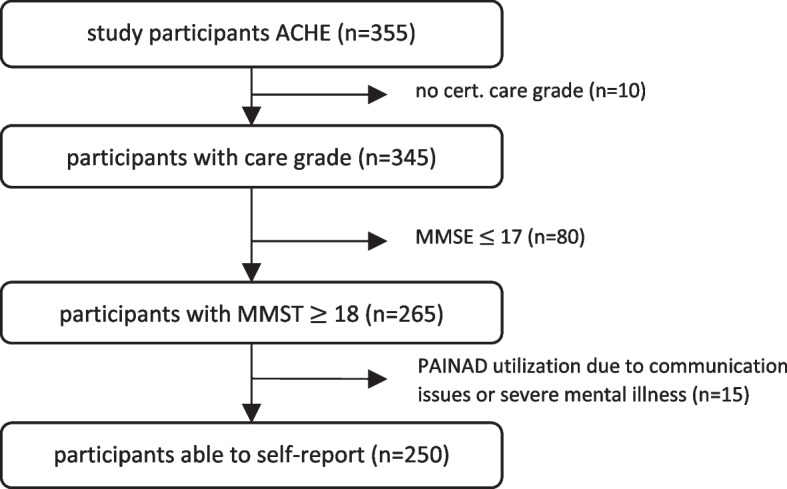
Participant flowchart

For this analysis, we included 250 participants (see Fig. 1). Inclusion criteria were a certified need of care according to the German Long-Term Care Insurance Act (*n *= 345) and, to ensure the ability to self-report, a score of ≥ 18 in the mini–mental state examination (MMSE ≥ 18, *n *= 265) [36]. In addition, participants whose pain situation was assessed with the help of the German version of the PAINAD scale [37, 38] were excluded (*n *= 15). Those participants were assessed as unable to self-report despite a MMSE of ≥ 18, mostly due to severe health issues (e.g., schizophrenia).

### Data collection

As primary outcomes, pain intensity and pain interferences were assessed with the German version of the Brief Pain Inventory (BPI), which uses numeric rating scales from 0-10 [39]. To determine whether the pain experienced should be considered bothersome and/or requires (further) treatment, various cut-off points on numeric scales are discussed in the literature [40–42] and a dichotomous assessment of the overall pain situation is also considered useful in pain research [43–45]. Consequently, two measures were chosen to assess the severity of the pain situation. Firstly, each participant was asked whether or not their pain situation was acceptable within the last four weeks [46]. Secondly, we assessed the pain situation with a composite measure based on the Pain, Enjoyment and General Activities (PEG) scale [47]. The PEG scale is a shortened instrument derived from the BPI, using the sum score of patients’ scoring of “average pain”, “enjoyment of life” and “general activity”, and defining a score of ≥12 as bothersome [42].

To assess differences between groups based on demographics, three age groups were formed (see Table 1). Care grades were originally assessed from 1 to 5, based on the German Long-Term Care Insurance Act [48], and then summarised in three levels from slight to considerable to severe impairment with the latter comprising care grades 3 to 5 (i.e., severe, more severe and severest level of impairment). Highest educational attainment was used as a proxy for SES and German school certificates and vocational and tertiary degrees were re-coded into ISCED-levels low, medium and high [49, 50].

**Table 1:** Demographics

Characteristics		Frequency	Percent
sex	female	172	68.8
	male	78	31.2
age groups	65-74	47	18.8
	75-84	112	44.8
	85 and older	91	36.4
level of impairment	slight impairment	38	15.2
	considerable impairment	146	58.4
	severe impairment	66	26.4
ISCED levels	low level of education	41	16.4
	medium level of education	163	65.2
	high level of education	45	18.0
	unknown level of education	1	0.4
	Total	250	100.0

NPI utilization was assessed with a structured list of 42 specific NPIs (e.g., hot water bottle, ice spray, massage, therapeutic exercise/physiotherapy) that was read out to each participant following the question “Which NPI was utilized for pain management within the last 4 weeks?”. After the dichotomous assessment of these 42 NPI, participants were able to name additional NPIs which were documented through free text fields. The structured list was developed during preceding projects [51] and it did not differentiate whether an NPI is considered active or passive. To analyse differences between participants who use active measures and/or passive ones, an appropriate categorization of NPIs was developed after data-collection.

A systematic literature search in December 2022 showed that many authors do not categorize NPIs by characteristics such as “active” or “passive” but rather by pain-related diagnosis [11], by individual lists of NPIs [e.g., 5, 18]) or by combining some of these characteristics with levels of evidence [e.g., 4, 19, 22]. Four sources were identified that differentiate between active and passive NPI [4, 6, 15, 21]. Cosio & Lin (2018) and Dunlop et al. (2013) agree that passive interventions should only be used adjunctive to active measures. In a recent systematic review, Skelly et al. [4] find “some support for clinical strategies that focus on “active” interventions as primary therapies, with “passive” interventions used in a more adjunctive or supplementary role”. Cosio and Lin [21] additionally name “transitional” as a third category, noting that “treatments [exist] in a continuum, with passive treatments being on one end and active on the other”. Based on the lists of examples from the literature, a categorization of all NPIs was proposed and discussed in a multidisciplinary team with experts from different backgrounds (i.e., physiotherapy, medical and nursing science).

### Data analysis

Descriptive statistics were used for demographics as well as for variables related to the utilization of NPIs. The distribution of numeric variables was determined by the Kolmogorov-Smirnov test. T-tests were conducted for normally distributed data. For not normally distributed data, the non-parametric Mann-Whitney U test or Kruskal-Wallis H test were used as appropriate. χ^2^ tests were used to determine differences between groups for categorical data. To determine whether the dichotomous assessment of the acceptability of participants’ pain situations is meaningful, we tested whether pain intensity and pain interferences scores are different in those who assess their pain as acceptable compared to those who perceive their pain as not acceptable. Logistic regression was performed to analyse the relationship between the utilization of certain NPIs and relevant demographic markers (including all predictor groups as covariates in a multivariate logistic model).

To answer the question whether the utilization of active or passive types of NPIs is associated with differences regarding participants’ pain situations, we assigned participants to mutually exclusive groups and analysed the data in two steps. Firstly, we used χ^2^ tests to check whether participants were more or less likely to assess their pain situation as acceptable or not depending on those groups. Secondly, we compared the means of certain pain intensity and pain interference measures, to assess whether there are differences between those who use certain types of NPIs (or none). The significance level was set at α = 0.05 with 95%-confidence intervals. Data analysis was conducted with IBM SPSS Statistics for Windows, version 27.0 (IBM Corp, Armonk, NY) and R (RStudio version 2023.06.0).

## Results

In total, 250 participants were included in this analysis; 172 (68.8%) of all participants were female and 78 (31.2%) male (see Table 1). The most common age group across both sexes was 75-84 years old, followed by the group of those 85 years and older. Only 15.2% of participants were considered slightly impaired (i.e., needing little support in daily life). The vast majority were considerably (58.4%) or severely impaired (26.4%). Across the three age groups, the levels of impairment were similarly distributed. Most participants (65.2%) had a medium level of education, most commonly in the form of vocational training. 16.4% were of low educational status while 18% indicated a high education level (i.e., at least a bachelor’s degree or similar).

The most commonly reported type of pain was low-back pain (82.4% of all participants), followed by pain caused by osteoarthritis (72.8%, *n *=243) and neuralgia-related pain (59.6% of all participants). Mean pain intensity indicates bothersome pain levels with x̅ = 5.37 for average pain (95%-CI 5.06 - 5.69) and x̅ = 7 for severest pain (95%-CI 6.65 - 7.34). Severest pain was x̅=6.34 (95%-CI 5.94 – 6.74) for those who perceive the situation as “acceptable” and with x̅=7.95 (95%-CI 7.6 – 8.3) clearly higher in those who specified their pain situation as “not acceptable” (*p* value = <0.001). Similarly, the average pain scores were indicated with x̅ = 4.85 (95%-CI 4.5 – 5.21) and x̅ = 6.2 (95%-CI 5.8 – 6.59, *p* value = <0.001) respectively. These differences were also observed with regard to pain interferences (*p* values <0.001 - 0.002). As a composite measure, the PEG score showed corresponding significant differences in means (x̅ “not acceptable” = 18.83 (95%-CI 17.77 – 19.9) vs. x̅ “acceptable” = 14.17 (95%-CI 13.02 – 15.33, *p* value <0.001). The group that described their pain situation as “acceptable” still indicated bothersome pain levels according to the PEG scale with an average sum score of more than 12.

The open question to gather additional NPIs resulted in 260 additional free-text answers. After removing duplicates and summarizing similar terms (e.g., “exercising”, “exercises with ball”, “sports group” was summarized as “exercise”), 140 unique terms remained. Together with the NPIs from the structured lists, these terms were recoded into 18 more general types of NPI and each type was assigned to one of four categories (see Table 2). The process of re-coding and categorization was accomplished through multiple rounds of interdisciplinary discussions and based on the current literature, differentiating between passive, transitional (to account for measures that may include active and passive approaches such as osteopathy) and active [4, 6, 21]. Active approaches were further divided into “movement based” and “cognitive” as this was deemed especially important in the context of the target group where frailty and restrictions in mobility are more common than in the general population. The mentioning of multiple measures from the same category was not counted. For instance, if someone stated that they distract themselves by reading, but also by playing cards, it was only reflected as the participant using distraction (NPI_distract = 1). Hence, if a specific NPI is recorded with “yes” it means that the participant uses at least one measure of that type.

**Table 2:** Categorization of NPIs

**Variable (recoded)**	**Label (recoded)**	**Categorization**
NPI_exercise	exercise (not guided by a physiotherapist)	active/movement based
NPI_occu	occupational therapy	active/movement based
NPI_physio	physiotherapy/therapeutic exercise	active/movement based
NPI_BF	biofeedback therapy	active/cognitive
NPI_distract	distraction	active/cognitive
NPI_edu	education	active/cognitive
NPI_psycho	psychological approaches	active/cognitive
NPI_relax	relaxation and mindfulness-based approaches	active/cognitive
NPI_acupuncture	acupuncture	passive
NPI_devices	devices, e.g., electric massage devices	passive
NPI_electro	electro therapy, e.g., TENS	passive
NPI_lymphdr	lymphatic drainage	passive
NPI_massage_th	massage by therapist	passive
NPI_position	positioning techniques to relief or prevent pain	passive
NPI_rest	rest or sleep to alleviate pain	passive
NPI_thermo	thermotherapy and compresses, e.g., ice spray, heat application	passive
NPI_comp	complementary therapies (other than acupuncture, e.g., osteopathy, aromatherapy, chiropractic, or music therapy)	transitional
NPI_massage_misc	massage; miscellaneous, e.g. self-massage with non-medicated oil	transitional

**Fig. 2:**
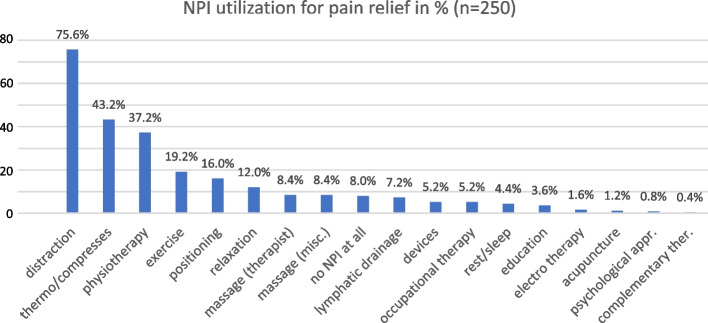
NPI utilization in % of total participants (*n* = 250)

In our sample, more than three-quarters (75.6%) of all participants utilize distraction (e.g., playing cards/board games, watching tv, listening to music, talking to relatives and/or neighbours) as a strategy to tackle their chronic pain. The second most common approach (43.2% of all participants) is thermotherapy which includes hot or cool packs as well as compresses or therapeutic measures like mud packs provided by physiotherapists. Physiotherapy (in the form of therapeutic exercise) is also widely used (37.2%) while exercise in general is utilized for pain management by less than a fifth (19.2%). Positioning and mindfulness or relaxation techniques were mentioned by 16% and 12%, respectively. Less commonly used NPIs include massage therapy, lymphatic drainage etc. which were each used by less than 10% (see Fig. 2). Only 20 participants (8%) used no NPI for pain relief.

**Fig. 3:**
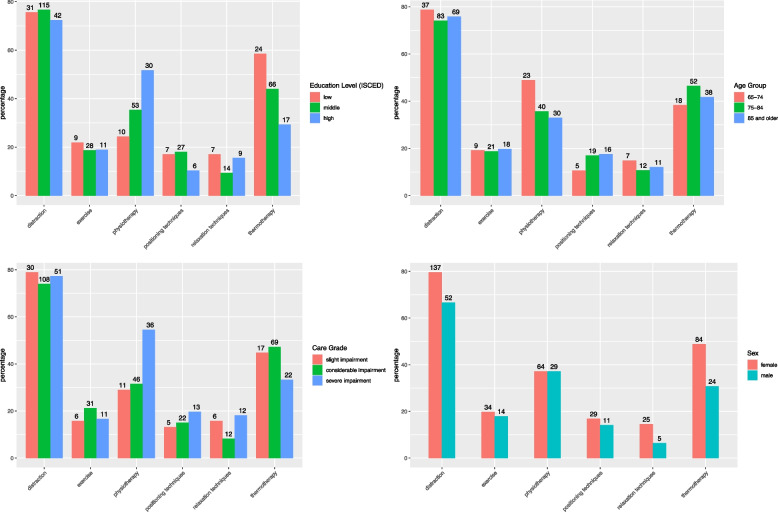
NPI utilization in % (absolute number on top of each bar) by education, age group, care grade, and sex

For the six most commonly used NPIs (all those mentioned by more than 10% of participants) we compared the proportionate utilization by age group, level of education, level of impairment and sex (see Fig. 3).

Most NPIs are similarly distributed across the three groups within the determinants education level, age, and level of impairment as well as between both sexes. Table 3 shows the adjusted odds ratios for those NPIs that graphically showed pronounced differences in proportions in at least one of those determinants (i.e., distraction, physiotherapy, and thermotherapy).

**Table 3:** Adjusted odds ratios for distraction, physiotherapy, and thermotherapy utilization

**Determinants for NPI utilization(*n *= 249)**	**Distraction**		**Physiotherapy**		**Thermotherapy**	
	**Odds ratio (95%-CI)**	***p* value**	**Odds ratio (95%-CI)**	***p* value**	**Odds ratio (95%-CI)**	***p* value**
**ISCED level**						
low level of education	Reference		Reference		Reference	
medium level of education	1.174 (0.515-2.673)	0.703	1.764 (0.785-3.965)	0.169	0.578 (0.283-1.180)	0.132
high level of education	1.048 (0.400-2.746)	0.924	3.259 (1.288-8.247)	**0.013**	0.374 (0.157-0.890)	**0.026**
**care grade**						
slight impairment	Reference		Reference		Reference	
considerable impairment	0.700 (0.292-1.682)	0.426	1.099 (0.494-2.449)	0.817	1.052 (0.503-2.197)	0.893
severe impairment	0.894 (0.333-2.401)	0.824	2.757 (1.148-6.622)	**0.023**	0.660 (0.284-1.535)	0.335
**age group**						
65-74	Reference		Reference		Reference	
75-84	0.675 (0.292-1.559)	0.357	0.596 (0.287-1.235)	0.164	1.219 (0.590-2.517)	0.593
85 and older	0.756 (0.318-1.795)	0.526	0.474 (0.222-1.013)	0.054	1.053 (0.499-2.225)	0.892
**sex**						
male	Reference		Reference		Reference	
female	2.055 (1.093-3.863)	**0.025**	1.358 (0.741-2.489)	0.322	1.809 (1.001-3.270)	0.050

Statistically significant differences are observed for distraction (between the sexes), physiotherapy (between highest and lowest levels of impairment and education respectively), and for the utilization of thermotherapy (between lowest and highest educational status and sex). There were no significant differences regarding levels of pain intensity or pain interference between the groups within the determinants sex, care grade, and educational level. Despite similar pain levels, it appears that females have two-fold increased odds (OR: 2.055, 95%-CI 1.093-3.863, *p* value = 0.025) to use the cognitive strategy of distraction when compared to men in our sample. More than half of all severely impaired participants utilized physiotherapy for pain management. Comparing those with a severe level of impairment to those with slight impairment, we found a positive association (OR: 2.757, 95%-CI 1.148-6.622, *p* value: 0.023) regarding physiotherapy utilization with more severe impairment. Differentiating by educational status, the observed group differences suggest that higher educational status is also positively associated with physiotherapy utilization. Compared to those with lower education, the odds of using physiotherapy are increased by three-fold for those with the highest level of education (OR: 3.259, 95%-CI 1.288-8.247, *p* value = 0.013). Conversely, those with high educational status were found to have decreased odds (OR: 0.374, 95%-CI 0.157-0.890, *p* value = 0.026) of using thermotherapy or compresses to alleviate their pain when compared to those with low educational status, meaning that the odds of utilizing thermotherapy or compresses for chronic pain are about three times higher for those with low educational status.

To assess potential differences between those who utilize (or not) active or passive (or a combination of both) NPIs, all participants were categorized into one of the following mutually exclusive groups:

1. active (movement), i.e., at least one active movement NPI (*n *= 126)
2. active (cognitive), i.e., at least one active cognitive, but no active movement NPI (*n *= 88)
3. passive, i.e., at least one passive, but no active NPI (*n *= 14)
4. no NPIs, i.e., no NPI utilization at all (*n *= 20)
5. unclear, i.e., only transitional/unclear (*n *= 2, excluded)

The vast majority of all study participants (85.6%) used some form of active NPI (cognitive or movement-based or both) and about half (50.4%) used at least one active movement approach to tackle their chronic pain. There was only a small group of participants who did not use any NPI (8%) or only passive approaches (5.6%). Those that use only “transitional” NPIs were excluded from this analysis (*n *= 2) as it cannot be determined to which group they belong.

**Fig. 4:**
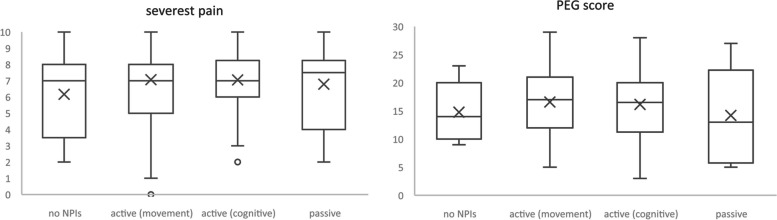
Boxplots of severest pain and PEG score dep. on NPI groups (‘x’ marking the mean in each group)

The odds of perceiving their pain situation as acceptable or not did not differ between those groups. Equally, no differences were found regarding pain levels and pain-related interferences, as can be seen in Figure 4 for perceived severest pain (0-10) and the PEG score (0-30) for each of these four groups (*n *= 248). When comparing the means, we observe slightly higher means in these two pain measures for those using active NPIs when compared to those who use none or only passive approaches; however, there are no clinically or statistically significant differences in means for these measures.

## Discussion

The aim of this analysis was to describe NPI utilization for chronic pain treatment in community-dwelling older adults (≥65 years) and, in a second step, active and passive approaches were distinguished to analyse potential group differences. Most older adults in our sample use active NPIs. Our data thereby indicate that many (consciously or unconsciously) follow recommendations to actively tackle chronic pain. When passive measures are utilized, they are commonly combined with active approaches which also aligns with current guidelines. However, the most recent primary care guideline for chronic pain management in Germany highlights the importance of active movement [18] and our results show that almost half of our sample does not use any movement-based approach.

Our results show no significant differences regarding the pain situation of those that use active vs. those that only use passive approaches. Since most participants in our sample use both, one possible explanation lies in small comparator groups (only 14 participants used only passive approaches and 20 used no NPI). The slight tendency to find higher pain scores in those that use active approaches might be related to people with higher pain intensity and more pain interferences trying out a greater variety of interventions to alleviate their pain. Conversely, those that do not suffer to a great extent might deem NPIs unnecessary or just use passive measures occasionally.

Therapeutic exercise provided by physiotherapists is considered an integral part of chronic pain management in Germany [18], especially for older populations [52]. The utilization of physiotherapy for pain management varies greatly across Europe [7]. One explanation for such variances might be rooted in nation-based differences regarding prescribing practices and cost coverage for physiotherapy. In Germany, physiotherapy is covered by the Statutory Health Insurance (SHI), requiring a small co-payment [53]. Breivik, Collett [7] found that 38% of German adults (not differentiating by age) with chronic pain have tried physiotherapy and, very similarly, the proportion in our sample is 37.2%. However, a more recent European study found that only 28.3% of older adults (≥55 years) with chronic pain use physiotherapy with this proportion reducing to 20.1% in those aged ≥85 [9]. Compared to the latter figures, our sample shows a relatively high physiotherapy utilization overall and especially in the oldest age group with 33.3% in the group ≥85 years. The association of care grade and physiotherapy might be rooted in the greater need for professionally guided movement for those that are severely impaired. For instance, people with disabilities were found to be significantly more likely to utilize physiotherapy than those without disability [54]. While level of impairment and disability are not equivalent, it appears logical that both may result in more prescriptions for physiotherapy as the need for such specialized therapy is higher.

Other NPIs, especially those that are considered part of the complementary alternative medicine (CAM) spectrum, are used less commonly in our sample when compared to European [9] and other German data [7]. For instance, acupuncture for pain relief was mentioned by 16% of German participants in one study [7], compared to only 1.2% in our sample. When considering manual body-based therapies, “the most commonly used CAM by older people with hampering pain” were massage therapy (17.9%) and osteopathy (7%) [9]. Breivik et al. [7] found that 46% of German adults with pain have tried massage to alleviate their symptoms. In our sample, only 8.4% indicated the use of therapeutic massage and not a single participant mentioned osteopathy. For the latter, one possible explanation may lie in the generally lower popularity of osteopathy in this age group in Germany. Data from 2016 shows that only 12% of older adults (≥50 years) have tried osteopathy [55]. Another explanation might be that osteopathy is not routinely covered by all health insurance providers under SHI regulations (as opposed to physiotherapy) [56].

Massage therapy, on the other hand, is covered for the treatment of many pain diagnoses by all insurance providers if prescribed by a physician and provided by an allied health professional such as a physiotherapist [56]. However, the number of massage sessions that can be prescribed is restricted in Germany [56] which might differ from other countries. Furthermore, our questionnaire asked for NPI-utilization within the last four weeks, which might be a possible reason for lower utilization-rates in our sample, compared to data from Europe [9], that reported NPI-utilization within the 12 months, and other age groups in Germany [7], which referred to any NPI-utilization in the past. In addition to these regulatory and study-related differences, financial restrictions of the German healthcare system might have played a role in prescription practices as well [57]. Similarly to massage therapy, acupuncture is covered by all insurance provides if it is prescribed by a physician for chronic low back pain or knee arthritis [56] but the uptake in our sample seems to be very low despite a high prevalence of these two pain-related diagnoses [58]. Overall, acupuncture utilization is considerably higher in Germany, with 38% of those ≥50 years of age indicating that they have used acupuncture in the past [59] and 16% (over all age groups) consider acupuncture as an effective measure to treat back pain [60]. One potential explanation for the low utilization-rate of acupuncture in our sample could lie within the specific national rules for public health insurance that allow acupuncture prescription only once a year for a maximum of ten to fifteen sessions [56]. Similarly to massage therapy, the four-week perspective of our questionnaire could also result in some people not reporting acupuncture as they have had their last session outside this specific time frame, further decreasing the utilization rate.

Evidence from pain research often shows a social gradient for the prevalence of pain [e.g., 61–66]. While our data shows no differences in severity of pain intensity or related interferences depending on SES, we found a difference in utilization of physiotherapy depending on educational status. When compared to those with low education, the odds of utilizing physiotherapy for chronic pain were found to be three times higher for those with the highest education level. Despite Germany’s universal health care system, social disparities in health outcomes and care utilization exist [67, 68]. Regarding the overall use of physiotherapy in Germany, the data show a social gradient depending on educational status [69], albeit not to the same extent as in our sample, and similar socioeconomic inequities regarding NPI utilization are found internationally [70–72]. Karran et al. [62] discuss how the “social” of the long established biopsychosocial model [73] is often limited to individual factors in the context of low-back pain “rather than the broader social conditions” which is likely to be true for to the management of chronic pain in general. While physicians might consider social factors like the direct social environment (e.g.: Is there someone to drive the patient to their physiotherapy session?), the educational status of an older person might be less commonly seen as an important factor when planning their chronic pain care.

To adequately address inequities in service utilization this issue needs to be viewed from the patients’ perspective as well. Barriers to NPI utilization for chronic pain care in older adults can be categorized into issues around awareness, appeal, and access [74]. With educational level as a proxy for SES, it becomes clear how each of these three areas might be influenced by education: Firstly, if older patients are not aware that physiotherapy might help with their pain condition, they will not request such care. Conversely, someone with higher educational attainment might be better informed and actively ask their physician for such prescriptions. Secondly, the appeal of certain measures can be influenced by previous experiences with the healthcare system which tend to be more negative for those of lower SES [75]. Hence, even if physiotherapy is offered, lower SES patients might be more likely to reject such treatment due to negative experiences in the past. Lastly, issues around access to care might pose a barrier, for example, when a lack of financial resources hinders utilization of physiotherapy even if awareness and appeal are not an issue. For instance, even if a patient is aware that they are entitled to physiotherapy, the existence of co-payments may still discourage utilization if not addressed.

Practical measures to address the barriers described could be focussed on awareness creation on the primary care providers’ side (e.g., by including information on SES-related differences in relevant vocational training and primary care guidelines) but also on the patients’ side (e.g., through plain language leaflets/brochures). Creating more transparency may also address issues around appeal and access. For instance, some patients with lower educational levels might not be aware that they are entitled to an exemption regarding the co-payments (e.g., based on a low income) or they might feel discouraged by the required forms and documents. Siegel and Busse [76] describe such processes as “bureaucratic” and criticise that it “must be repeated annually”, which clearly affects people with low education disproportionately and could be addressed by providing more individualized guidance through care providers. However, more research is needed to better understand the mechanisms behind the existing inequalities in healthcare utilization in this specific population.

Overall, our findings indicate that utilization rates of active movement NPIs could be increased in this target group. Primary care providers should recommend such active approaches to older patients, always considering the individual patient’s abilities and preferences, of course. Appropriate forms of active movement might not only alleviate pain but may also help to maintain mobility more generally, which can positively affect older peoples’ overall quality of life.

### Limitations

Due to the cross-sectional design of our study, it is not possible to establish causal associations. Longitudinal studies are needed to examine the effectiveness of NPIs for chronic pain management in older community-dwelling adults with specific care needs since this population group is rapidly growing and was often not represented in previous studies [4, 21, 71]. Since it was not assessed how often and for how long participants performed each NPI it is unclear whether there might be differences based on the extent to which NPIs are used with regard higher or lower pain levels or more or less severe pain interferences.

While we included relevant sociodemographic parameters as covariates in our logistic model, it was not possible to adjust for other possible confounders (e.g., certain pain diagnoses or use of certain pain medication) because sample size was not sufficient. Finally, our sample consisted of mostly Caucasian older adults (≥65 years) living in the city of Berlin, Germany, and results are therefore not generalizable to populations differing in ethnicity or age or to older adults living in more rural areas.

## Conclusion

Most community-dwelling older adults use active NPIs, but utilization of active movement could be increased as its importance is emphasized in recent guidelines. Physiotherapy in the form of therapeutic exercise is an appropriate variant of active movement interventions for this highly vulnerable target group but there appears to be a social gradient in its utilization for chronic pain management. Patients with lower educational status might be less demanding or even not aware that they are entitled to such therapy options at a low cost. To tackle the unequal provision of healthcare services for chronic pain, primary care providers need to be aware of such socioeconomic differences and researchers and policy makers should address them accordingly, for instance when developing research protocols or new guidelines.

## Data Availability

The data that support the findings of this study are not openly available due to reasons of sensitivity and are available from the corresponding author upon reasonable request. Data are located in controlled access data storage at Charité - Universitätsmedizin Berlin.
